# Comparison of accuracy for hip-knee-ankle (HKA) angle by X-ray and knee motion analysis system and the relationships between HKA and gait posture

**DOI:** 10.1186/s12891-023-06437-3

**Published:** 2023-06-03

**Authors:** Hui Zhang, Yanan Chen, Huiquan Jiang, Wenqing Yan, Yuanming Ouyang, Wei Wang, Yaru Liu, Ying Zhou, Shiyi Gu, Hong Wan, Axiang He, Yanjie Mao, Wanjun Liu

**Affiliations:** 1 Department of Joint Surgery, Shanghai Jiao Tong University Affiliated Sixth People’s Hospital, 222 West Huanhu Third Road, Pudong New Area, Shanghai, 201306 China; 2 College of Food Science and Nutritional Engineering, China Agricultural University, Beijing, 10083 China; 3 College of Food Science and Technology, Shanghai Ocean University, No. 999, Hucheng Ring Road, Pudong New Area, Shanghai, 201306 China; 4 College of Fisheries and Life Science, Shanghai Ocean University, No. 999, Hucheng Ring Road, Pudong New Area, Shanghai, 201306 China

**Keywords:** Hip-knee-ankle angle, Gait analysis, 3D knee kinematics, Knee osteoarthritis, Knee surgery

## Abstract

**Background:**

The lower limb mechanical axis was used to assess the severity of knee osteoarthritis (KOA) with varus/valgus deformity and the accuracy of targeted lower limb alignment correction after operation by conventional X-rays. There are lots of parameters to assess the gait in elder patients such as velocity, stride length, step width and swing/stance ratio by knee joint movement analysis system. However, the correlation between the lower limb mechanical axis and gait parameters is not clear. This study is aimed at obtaining the accuracy of the lower limb mechanical axis by the knee joint movement analysis system and the correlation between the lower limb mechanical axis and gait parameters.

**Methods:**

We analysed 3D knee kinematics during ground gait of 99 patients with KOA and 80 patients 6 months after the operations with the vivo infrared navigation 3D portable knee joint movement analysis system (Opti-Knee®, Innomotion Inc, Shanghai, China). The HKA (Hip-Knee-Ankle) value was calculated and compared to X-ray findings.

**Results:**

HKA absolute variation after the operation was 0.83 ± 3.76°, which is lower than that before the operation (5.41 ± 6.20°, p = 0.001) and also lower than the entire cohort (3.36 ± 5.72). Throughout the cohort, a significant correlation with low coefficients (r = -0.19, p = 0.01) between HKA value and anterior-posterior displacement was found. In comparing the HKA values measured on the full-length alignment radiographs and 3D knee joint movement analysis system (Opti-Knee), there was a significant correlation with moderate to high coefficients (r = 0.784 to 0.976). The linear correlation analysis showed that there was a significant correlation between the values of HKA measured by X-ray and movement analysis system (R^2^ = 0.90, p < 0.01).

**Conclusions:**

Data with equivalent results as HKA, the 6DOF of the knee and ground gait data could be provided by infrared navigation based 3D portable knee joint movement analysis system comparing with the conventional X-rays. There is no significant effect of HKA on the kinematics of the partial knee joint.

## Introduction

Knee osteoarthritis (KOA) is a common joint disorder especially in the elderly and its incidence is rising [1]. There are various methods for evaluating KOA, mainly scoring systems [2], imaging [3, 4] and reference standards [5]. Among them, radiographic film is the simplest, cheapest, and the main method for the diagnosis and evaluation of KOA. Lower limb alignment is an eminent indicator of load transmission. The most classic method of assessing the alignment of lower limbs is to measure the hip-knee-ankle angle (HKA), which is measured by full-length radiographs of the lower limbs covering the entire hip, knee, and ankle joints in the weight‐bearing position [6]. When arthritis is developed, the arithmetic HKA can predict the constitutional alignment [7]. Phenotyping of HKA in young non-osteoarthritic knees provides better understanding of native alignment variability [8]. The HKA angle is associated with the subchondral trabecular bone microarchitecture which is related to KOA severity [9]. HKA demonstrates super diagnostic validity concerning medial and lateral joint space narrowing [10]. Standing HKA radiographs have been recognized as a key component of successful surgeries. Theoretically, the HKA angle is highly suitable as a preoperative planning parameter for High tibial osteotomy (HTO) to ensure optimal post-operative alignment [11]. It is used in a plane preoperative planning of HTO to predict osteotomy depth, open height and correction angle according to the magnitude of deformity [12]. HKA was compared between neutral mechanical alignment and residual varus after total knee arthroplasty (TKA) to calculate the satisfactory survival rate [13].

However, several studies have found an insignificant relationship between HKA and TKA survivorship in recent years [13–16]. Indeed, HKA cannot be the only indicator to evaluate the surgical effect, survival rate and patient satisfaction. HKA is not representative of the dynamic loading occurring during gait, unlike the gait analysis which is a promising technique for assessing the biomechanical changes of the patients’ lower limbs objectively. There are lots of parameters to assess the gait in elder patients with KOA such as velocity, stride length, step width, swing/stance ratio, the smoothness of gait activity, and maximum angular velocity [17]. Gait analysis can also be used to evaluate early effectiveness after TKA including knee flexion range, stride length, cadence, and compensatory hip and ankle rotation range [18]. The classification model along with biomechanical features can be used as an extra tool for objective and repeatable KOA diagnosis, reflecting the key gait features of different grades of KOA and representing joint function from the early to the final stage of the disease [19]. To date, there have been no reports on the six degrees of freedom (6DOF) of the knee joint data to compare the effect of the lower limb mechanical axis for patients with KOA.

The goal of the present study was two-fold: (1) to analyse HKA throughout the gait cycle by infrared-based navigation three-dimensional (3D) knee joint movement analysis system and obtain it’s accuracy, (2) to find out if the 6DOF of the knee joint data is related to lower limb mechanical axis. We hypothesized that the 3D knee joint movement analysis system could measure HKA, and that 6DOF of the knee joint data varies with HKA.

## Materials and methods

### Subjects

Demographic and anthropometric characteristics of all the groups are summarized in Table 1. 99 patients with KOA and 80 patients about six months after surgery (i.e. TKA, Unicompartmental Knee Arthroplasty (UKA), HTO, Distal Femoral Osteotomy (DFO) and Patellofemoral Arthroplasty (PFA)) from January 2019 to December 2019 in the Department of Joint Surgery at our hospital were selected. Based on different HKA angles, preoperative patients were divided into group A (-6°- 6°) and group B (< -6° and > 6°); patients six months after operation were divided into group C (-3°- 3°) and group D (< -3° and > 3°). The exclusion criteria for patients included, (1) Patients without history of major trauma, surgery or knee-related symptoms. (2) Patients with rheumatoid arthritis, ankylosing spondylitis, and other autoimmune diseases. (3) Patients with knee joint tumour, infection, severe osteoporosis, and other diseases affecting osteotomy healing. (4) Patients with concurrent severe flexion contracture deformities. This study was conducted based on the protocol approved by the Institutional Review Board. Patients who participated in the experiment joined voluntarily and fully understood the clinical trial protocol.

**Table 1:** Characteristics of the selected participants

	Pre		p	Post		p
	Group A (-6°- 6°)	Group B (< -6° and > 6°)		Group C (-3°- 3°)	Group D (< -3° and > 3°)	
Sex (female/male)	31/18	38/12	0.19	38/10	27/5	0.77
Age (y)	65.98 ± 7.45	64.98 ± 10.40	0.58	63.94 ± 7.19	63.22 ± 5.28	0.63
Height (cm)	160.10 ± 6.60	158.94 ± 7.99	0.43	159.81 ± 8.27	159.03 ± 8.27	0.68
Weight (kg)	66.67 ± 9.50	67.80 ± 10.38	0.58	66.13 ± 10.27	65.31 ± 11.27	0.74
BMI (kg/m^2^)	26.04 ± 3.67	26.83 ± 3.59	0.28	25.92 ± 3.72	25.70 ± 2.97	0.78

### Admission check and surgical method

After admission, routine preoperative examinations (CRP, ESR, liver and kidney function, electrocardiogram, etc.) and relevant examinations including bone density, CT and MRI scan of knee joints, full-length radiographs and short knee radiographs have been checked. According to the patients’ symptoms, signs, bone density, X-ray, CT and MRI examinations, different patients received the corresponding surgical methods:

(1) TKA: knee osteoarthritis grade of Kellgren-Lawrence Grade III or above; Multiple compartment osteoarthritis; medial articular surface “bone to bone”; severe patellofemoral joint degeneration;

(2) UKA: Single-compartment osteoarthritis of the knee joint; without severe line of force; The anterior cruciate ligament (ACL) and collateral ligament are in good condition; Intact or mild degeneration of cartilage in the contralateral compartment and patellofemoral joint;

(3) HTO: The patients who are younger than 60 years and require large activities; Single-compartment osteoarthritis patients with poor knee joint force; No or mild osteoarthritis in the tibiofemoral and patellofemoral joints; No osteoporosis; Varus deformity originates from the tibial side;

(4) DFO: Varus deformity originates from the femoral side;

(5) PFA: Patients with simple and severe patellofemoral osteoarthritis; without coaxial bone distortion, poor alignment of the lower limbs or large varus and valgus angles.

### Gait analysis device

3D knee joint movement analysis system, as the integrated system for dynamic and real-time examination of knee joint movement function, could provide doctors with the 6DOF of the knee joint quickly and accurately. It could measure the stability of knee joints under different states of motion such as flat walking, squatting, uphill and rotation to evaluate the function of knee joints before and after the operation, and it will generate inspection reports immediately. These reports, combined with X-ray/ CT/ MRI and other imaging examinations of joint structure, helped doctors evaluate the knee joint movement function of subjects objectively.

### Experimental procedure and functional assessment

**Fig. 1:**
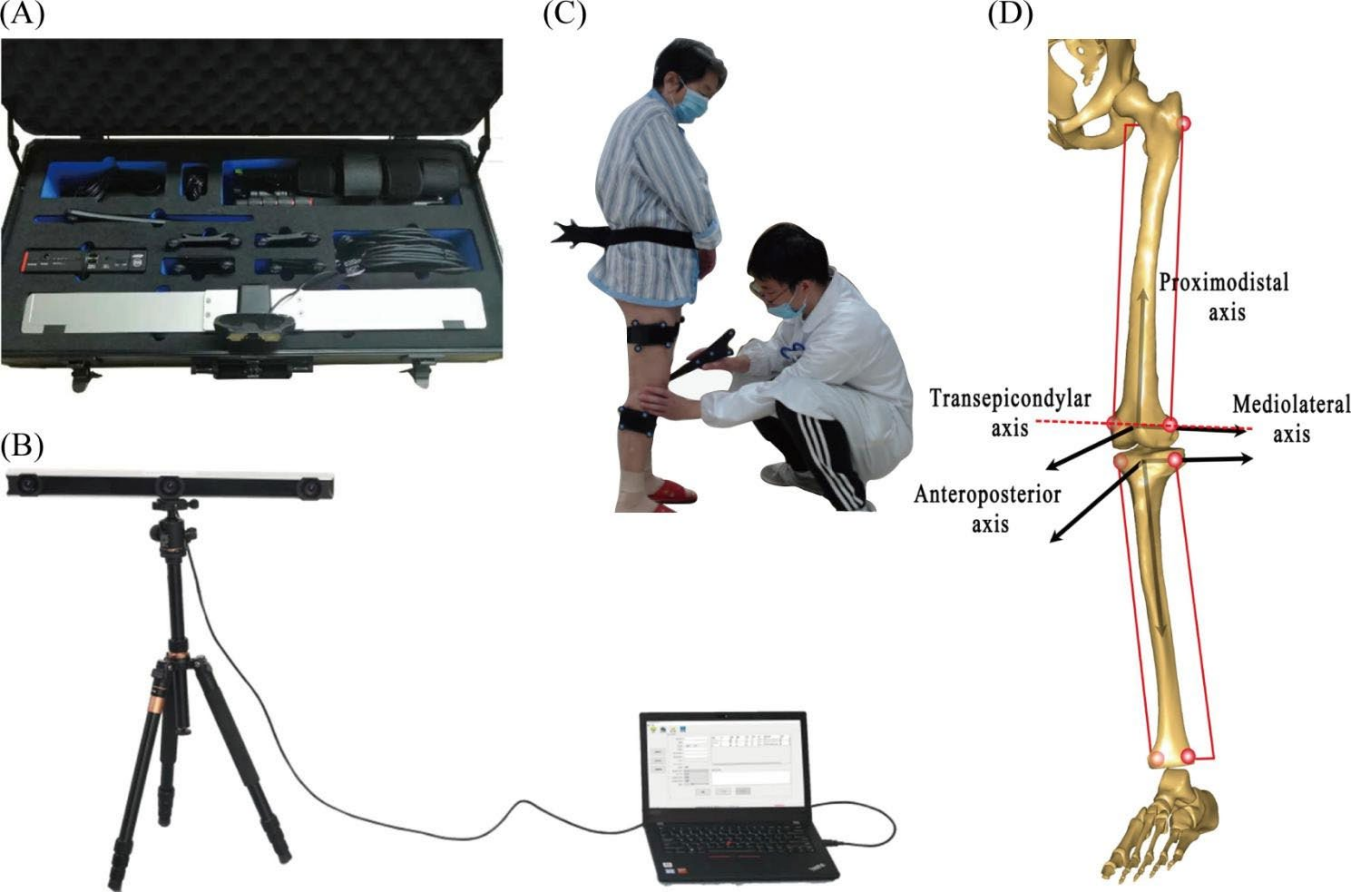
**(A)** Infrared navigation 3D portable knee joint movement analysis system. **(B)** Integrated dual stereo infrared camera and computer. **(C)** Identifying the femoral and tibial anatomical landmarks, the illustrated probe is pointing to the medial tibial plateau. **(D)** Definition of local femur and tibia coordinate systems

A vivo infrared-based navigation three-dimensional knee joint movement analysis system (Opti-Knee®, Innomotion Inc, Shanghai, China) was used to record and analyse the kinematic data of the knees in 6DOF of both knees one day before surgery and six months after surgery (Fig. 1A).

Before the test, the doors and windows were closed and curtains were drawn to block external light. All luminous objects in the room were removed to avoid the interference of external interference on the data. The subjects took off the pants and fully exposed both lower limbs. The subjects were told to raise their head and chest, look straight ahead, maintain a standard standing posture, with arms akimbo to prevent the markers from being blocked, and then performed the test system calibration. Two rigid plates, each with four infrared light-reflecting markers (OK_Marquer; Innomotion), were attached to the thighs and shanked with bandages. The 3D motion of the rigid plates was tracked by a stereo binocular infrared camera at a frequency of 60 Hz (Fig. 1B). A hand-held digitizing probe, with four infrared reflective light-reflecting markers, was used to identify femoral and tibial landmarks on the femur and tibia (Fig. 1C). Femoral and tibial landmarks included the trochanter major, condylus lateralis, condylus medialis, medial tibial plateau, lateral tibial plateau, medial malleolus, and lateral malleolus (Fig. 1D).

When the patients walked freely and normally, an integrated synchronous infrared camera was collecting walking videos and gait data at a frequency of 60 frames per second for the 60s to identify gait cycles and then calculate 6DOF in real time. On the standing full-length X-ray film of both lower limbs, mark the center of the hip joint, the center of the knee joint, and the center of the ankle joint. The HKA is the acute medial angle formed by the mechanical axes of the femur and the mechanical axes of the tibia. The HKA measured by weight-bearing full-length X-ray images and 3D knee joint movement analysis system were compared.

### Statistical analysis

All data processing and statistical analyses were undertaken with sigmaplot 14.0 (Systat Software Inc., San Jose, CA, USA) and SPSS 26.0 (IBM Corp., Armonk, NY, USA), respectively. Continuous variables following normal distribution were expressed as means (SD) with paired t-tests. Pearson correlation coefficients between 6 DOF and HKA values were analyzed for the whole cohort and the different groups separately. A difference of p < 0.05 was considered to be statistically significant.

## Results

### 3D knee kinematic alterations in ground gait

Among the 179 knees analysed in the present study, 99 (56%) were preoperative and 80 (44%) were post-operative. Comparisons of 3D knee kinematic parameters in the gait events between pre- and post-operation are shown in Table 2. The mean ± standard deviation (SD) of 6DOF of the subjects during the ground gait is shown in Table 2. Differences in HKA angles were not statistically significant for 6DOF values (p > 0.05).

**Table 2:** Comparison of the whole cohort and the various groups HKA and 6DOF during a mean gait cycle

		HKA	6 DOF					
		(X-ray)	Flexion/extension angle	Varus/valgus angle	Internal/external rotation	Anterior-posterior displacement	Superior-inferior displacement	Medial-lateral displacemet
Whole	Pre	5.41 ± 6.20	42.75 ± 12.41	8.22 ± 5.11	13.94 ± 6.29	1.91 ± 2.06	1.72 ± 2.09	1.00 ± 0.52
	N = 99	[-12.11-22.46]	[10.6–76.8]	[0-34.2]	[2.6–30.8]	[0.4–20.7]	[0.4–19.5]	[0.3–3.1]
	Post	0.83 ± 3.76	45.51 ± 11.11	8.01 ± 3.98	13.55 ± 5.65	2.08 ± 1.04	1.50 ± 0.55	0.96 ± 0.48
	N = 80	[-11.97-10.97]	[10.8–62.6]	[0-19.7]	[3.6–28.3]	[0.6–5.5]	[0.5-3]	[0.3–2.5]
	p	0.00**	0.12	0.76	0.66	0.51	0.37	0.58
Pre	A	2.04 ± 2.48	44.97 ± 10.31	7.82 ± 3.57	14.10 ± 6.77	1.77 ± 0.80	1.50 ± 0.54	0.97 ± 0.43
	n = 52	[-5.12-5.75]	[12.4–62.2]	[0-16.9]	[2.6–29.7]	[0.4–4.4]	[0.4–2.6]	[0.3–2.5]
	B	10.58 ± 5.41	40.29 ± 14.09	8.67 ± 6.41	13.77 ± 5.78	2.06 ± 2.87	1.96 ± 2.98	1.04 ± 0.60
	N = 47	[-7.39-22.46]	[10.6–76.8]	[0-34.2]	[3.6–30.8]	[0.5–20.7]	[0.4–19.5]	[0.3–3.1]
	p	0.00**	0.06	0.42	0.80	0.50	0.28	0.51
Post	C	0.11 ± 1.65	46.08 ± 11.92	8.18 ± 4.02	13.34 ± 6.09	1.98 ± 1.04	1.55 ± 0.56	1.01 ± 0.46
	n = 48	[-2.86-2.98]	[10.8–60.5]	[0-19.7]	[4.4–28.3]	[0.6–5.5]	[0.5-3]	[0.3–2.5]
	D	2.91 ± 5.02	44.66 ± 9.87	7.75 ± 3.98	13.85 ± 5.00	2.22 ± 1.05	1.44 ± 0.52	0.89 ± 0.50
	n = 32	[-7.59-11.12]	[20.9–62.6]	[2.2–18.1]	[3.6–28.1]	[0.6-5]	[0.5–2.9]	[0.5-3]
	p	0.31	0.58	0.64	0.69	0.33	0.38	0.28

* Statistically significant difference (P < 0.05), paired t test, two-tailed

**Fig. 2:**
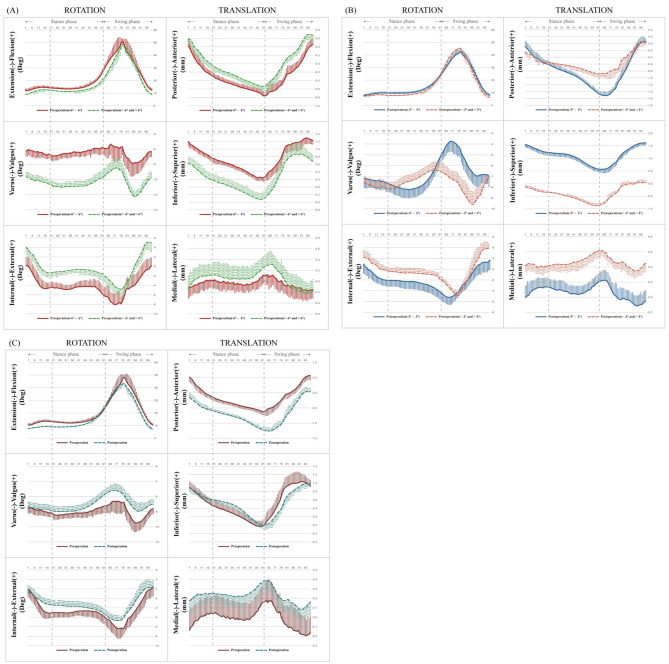
The 3D knee kinematic curves, including rotations and translations during ground walking gait, pre-operation **(A)**, post-operation **(B)** and the whole **(C)**. The ensemble of each runner was normalized from the heel strike to the next heel strike of the same foot as a gait cycle. The solid (dashed) curves and the lines above (below) them represent the mean and the SD (variability of these cycles) of the gait cycle for two groups respectively

The changes in the range of motion during gait are shown in Fig. 2. In both the stance phase and the swing phase, knee kinematic changes were similar in the groups (p > 0.05). Common traits were discovered by grouping and comparative analysis based on the difference in HKA angles: A larger difference in HKA angle results in greater distal-proximal displacement but less external/internal angle, anterior-posterior displacement and medial-lateral displacement (Fig. 2 AB). The superior-inferior displacement of the post-operation was greater than the pre-operation during the stance phase, whereas the value of the post-operation was inferior to the pre-operation in the swing phase (Fig. 2C).

The postoperative absolute variation (0.83 ± 3.76° [-11.97° − 10.97°]) was significantly lower than the preoperative value (5.41 ± 6.20° [-12.11° − 22.46°], p = 0.001). Only the preoperative value of (42.75 ± 12.41 [10.6–76.8]) is lower than the postoperative value (45.51 ± 11.11 [10.8–62.6]). For five of the remaining 6DOF, the absolute variations of pre-operation were higher than the post-operation values. Grouped according to the value of distinct HKA, there was no statistically significant difference between the 6DOF of each group. Table 3 shows the Pearson correlation coefficients between 6DOF and HKA values for the whole cohort and the various groups. When comparing flexion/extension angle and HKA with Group A, significant differences were found (r = 0.29, p = 0.042). There were significant correlations with low coefficients (r = -0.19, p = 0.01; r = -0.31, p = 0.03) between HKA value and anterior-posterior displacement for the whole cohort and group C.

**Table 3:** Pearson correlation coefficients between HKA and 6DOF for the whole cohort and the various groups

		HKA	6 DOF					
			Flexion/extension angle	Varus/valgus angle	Internal/external rotation	Anterior-posterior displacement	Superior-inferior displacement	Medial-lateral displacement
Whole	Pearson		-0.11	0.14	0.00	-0.19*	-0.05	-0.16
N = 179	Sig		0.13	0.06	0.99	0.01	0.52	0.84
Pre	Pearson		-0.10	0.20	-0.20	-0.22	-0.08	0.17
N = 99	Sig		0.32	0.05	0.85	0.03	0.45	0.87
post	Pearson		-0.19	0.01	-0.01	-0.06	-0.16	-0.15
N = 80	Sig		0.87	0.95	0.98	0.57	0.17	0.18
A	Pearson		0.29*	-0.74	0.12	0.10	0.24	0.05
N = 49	Sig		0.042	0.61	0.41	0.50	0.09	0.71
B	Pearson		-0.09	0.23	-0.06	-0.31*	-0.17	-0.04
N = 50	Sig		0.52	0.10	0.69	0.03	0.25	0.78
C	Pearson		0.17	0.03	-0.05	-0.06	-0.10	0.14
N = 48	Sig		0.24	0.84	0.72	0.69	0.52	0.34
D	Pearson		-0.10	0.02	0.00	-0.19	-0.20	-0.26
N = 32	Sig		0.58	0.91	0.99	0.31	0.28	0.15

* correlation is significant at the 0.05 level (2-tailed)

**Fig. 3:**
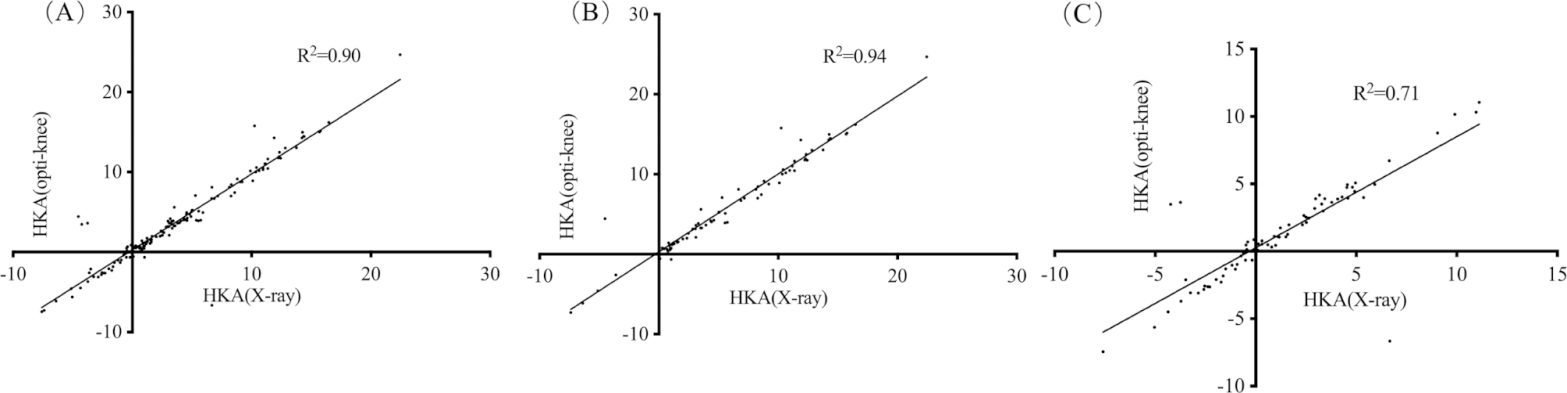
Linear correlation between the values of HKA measured on the full-length alignment radiographs (X-ray) and 3D knee joint movement analysis system (Opti-Knee), the whole (A), pre-operation (B) and post-operation (C)

There were significant correlations with moderate to high coefficients (r = 0.784 to 0.976) between the comparisons of HKA measured on long leg alignment radiographs and optical motion capture system (Opti-Knee) in Table 4. In the linear correlation analysis, R^2^ = 0.90 indicated that the model fitted well; the p-value of the analysis of variance of the linear regression model was 0.000, indicating the statistical significance between the independent variable “the values of HKA measured on the full-length alignment radiographs” and the dependent variable “the values of HKA measured on the 3D knee joint movement analysis system”. The preoperative patients’ data and postoperative values were (R^2^ = 0.94, p < 0.01) and (R^2^ = 0.71, p < 0.01) in Fig. 3.

**Table 4:** Pearson correlation coefficients between HKA measured by X-ray and Opti-Knee

	Pre			Post			Whole
	Group A	Group B		Group C	Group D		
X-ray	2.04 ± 2.48	10.58 ± 5.41	5.92 ± 5.89	0.11 ± 1.65	2.91 ± 5.02	1.27 ± 3.71	3.68 ± 5.46
	[-5.12-5.75]	[-7.39-22.46]	[-7.39-22.46]	[-2.86-2.98]	[-7.59-11.12]	[-7.59-11.12]	[-7.11-22.46]
Opti-knee	2.21 ± 2.25	10.68 ± 5.61	6.07 ± 5.91	0.17 ± 1.75	2.92 ± 4.86	1.31 ± 3.65	3.76 ± 5.47
	[-4.60-7.10]	[-7.27-24.68]	[-7.28-24.68]	[-3.10-3.90]	[-7.45-11.05]	[-7.45-11.05]	[-7.45-24.68]
Pearson	0.784**	0.976**	0.971**	0.970**	0.789**	0.84**	0.948**
Sig	0.000	0.000	0.000	0.000	0.000	0.000	0.000

**correlation is significant at the 0.01 level (2-tailed)

## Discussion

No significant difference was observed between values of HKA measured on the full-length alignment radiographs and 3D knee joint movement analysis system (Opti-Knee). It was demonstrated that the knee motion analysis might offer data with equal results to HKA.

The knee motion analysis uses the method of labeling in the body surface, recording the landmarks and calculating the distances between landmarks. By recording the femoral and tibial landmarks labeled by a hand-held digitizing probe with four infrared landmarks in each frame of the gait, the 3D positions were then measured as the distances between the landmarks in the femoral and tibial coordinate system. This method has certain consistency and credibility, which makes it different from other speculation methods. For example, standardized anteroposterior knee radiographs are insufficient to assess lower limb alignment of post-operative TKA [20].

However, no significant difference was found between tested groups regarding the 6DOF based on data collected in Table 2. These outcomes are consistent with the findings in other relevant research literature available at present. It was found by Yan W that no significant statistical differences between the anterior cruciate ligament reconstruction knees and the corresponding contralateral normal knees were observed regarding the 5DOF [21]. Also, one other study had comparable results. According to Peixoto JG, there was no correlation between older women with bilateral knee and asymptomatic controls in relation to the step length and single support phase between lower limbs [22]. These three results collectively explained the insignificant difference in gait posture among different groups of mechanical axes after surgery. In addition to the above conclusion, there were significant correlations with low coefficients (r = -0.19, p = 0.01) only between anterior-posterior displacement and the HKA values for the whole cohort as shown in Table 3. This meant minor varus/ valgus alignment does not compromise gait posture; the 6DOF cannot be the indicator to evaluate the alignment of lower limbs.

In other related post-operation studies, the 6DOF in ground gait suggested that varied static HKA does not correspondingly predict kinematics [23]. It has been shown that minor varus alignment does not compromise the mid-to long-term outcome of a medial UKA with no more than 7° of varus by analyzing medial fixed-bearing UKAs [24]. This can be explained by that the post-operative mechanical axis has little effect on post-operative results according to IKS function scores or muscle strength et al. in previous studies [15]. Clément J noted that lower limb radiographic measures of coronal alignment have limited value for predicting dynamic measures during gait [25]. Similar results have been reported that no obvious benefit for coronal alignment outcomes in a systematic review and Meta-analysis on PSI knee arthroplasty [16]. Interestingly, comparing kinematic alignment TKA patients with mechanical alignment TKA patients, kinematic alignment in TKA reproduces normal gait better than mechanical alignment [26].

Recent studies have shown that the single mechanical alignment often leads to significant anatomical modifications with a wide range of complex collateral ligament imbalances, which cannot be corrected by releasing collateral ligament [13, 26, 27]. A possible explanation for these unsatisfactory results may be related to the functional outcome of knee arthroplasty and osteotomy. There were significant differences in walking speed, cadence and stride length between UKA patients and healthy controls during level walking which means UKA cannot completely restore normal gait patterns during level walking clinically [28]. These outcomes were in agreement with the outcome of TKA. The knee kinematics during gait in the TKA group improved. However, it could not fully reach the level of the healthy control group [29]. In comparison, neither type of knee arthroplasty restored knee kinematics to those of the non-operated side [30]. However, a more natural loading pattern can be achieved with UKA as compared to TKA [12]. Some gait features were also found to differ between post-HTO subjects and controls [31]. By using a dynamic metric of everyday activities, distinct gait differences between various arthroplasty types were established [12].

Lower limb radiographic measures in the previous studies have limited value for predicting HKA, which revealed the irreplaceability of HKA. For patients, while measuring HKA by the 3D knee movement analysis system, the radiographic exposure was reduced. It reported the HKA visually so that clinicians no longer need to measure it on full-length standing radiographs of the patient.

There were several limitations of this study. Firstly, there may be systematic errors in making comparisons for the HKA of different individuals for both pre- and post-operation measurements. Secondly, we only compared gait posture and HKA in about six months after operations, with a lack of longitudinal follow-up. Thirdly, we only compared knee kinematic changes in patients without measuring the healthy group as control.

## Conclusion

In comparison with the conventional X-rays, the 3D portable knee joint movement analysis system could provide data with equivalent results for HKA and ground gait data simultaneously. There is no significant effect of HKA on the kinematics of the partial knee joint.

## Data Availability

The datasets during and/or analyzed during the current study available from the corresponding author on reasonable request.
